# Cancer diagnoses after emergency GP referral or A&E attendance in England: determinants and time trends in Routes to Diagnosis data, 2006–2015

**DOI:** 10.3399/bjgp19X705473

**Published:** 2019-08-28

**Authors:** Annie Herbert, Gary A Abel, Sam Winters, Sean McPhail, Lucy Elliss-Brookes, Georgios Lyratzopoulos

**Affiliations:** MRC Integrative Epidemiology Unit, University of Bristol, Bristol; formerly at Epidemiology of Cancer Healthcare and Outcomes (ECHO) Group, University College London, London; National Cancer Registration and Analysis Service (NCRAS), Public Health England, London.; University of Exeter Medical School (Primary Care), Exeter; National Cancer Registration and Analysis Service (NCRAS), Public Health England, London.; National Cancer Registration and Analysis Service (NCRAS), Public Health England, London.; National Cancer Registration and Analysis Service (NCRAS), Public Health England, London.; National Cancer Registration and Analysis Service (NCRAS), Public Health England, London.; Epidemiology of Cancer Healthcare and Outcomes (ECHO) Group, University College London, London; National Cancer Registration and Analysis Service (NCRAS), Public Health England, London.

**Keywords:** early diagnosis, emergencies, patients, population groups, referral and consultation

## Abstract

**Background:**

Diagnosis of cancer as an emergency is associated with poor outcomes but has a complex aetiology. Examining determinants and time trends in diagnostic routes can help to appreciate the critical role of general practice over time in diagnostic pathways for patients with cancer.

**Aim:**

To examine sociodemographic, cancer site, and temporal associations with type of presentation among patients with cancer diagnosed as emergencies.

**Design and setting:**

Analysis of Routes to Diagnosis data, 2006–2015, for patients with cancer in England.

**Method:**

The authors estimated adjusted proportions of emergency presentation after emergency GP referral (GP-EP) or presentation to accident and emergency (AE-EP), by patient sex, age, deprivation group, and year of diagnosis using multivariable regression.

**Results:**

Among 554 621 patients presenting as emergencies, 24% (*n* = 130 372) presented as GP-EP, 62% as AE-EP (*n* = 346 192), and 14% (*n* = 78 057) through Other-EP sub-routes. Patients presenting as emergencies were more likely to have been GP-referred if they lived in less deprived areas or were subsequently diagnosed with pancreatic, gallbladder, or ovarian cancer, or acute leukaemia. During the study period the proportion and number of GP-EPs nearly halved (31%, *n* = 17 364, in 2006; 17%, *n* = 9155 in 2015), while that of AE-EP increased (55%, *n* = 31 049 to 68%, *n* = 36 868).

**Conclusion:**

Patients presenting as emergencies with cancers characterised by symptoms/signs tolerable by patients but appropriately alarming to doctors (for example, pancreatic cancer manifesting as painless jaundice) are over-represented among cases whose emergency presentation involved GP referral. Reductions in diagnoses of cancer through an emergency presentation likely reflect both the continually increasing use of 2-week-wait GP referrals during the study period and reductions in emergency GP referrals.

## INTRODUCTION

Around one in five patients with cancer are diagnosed as an emergency, which is associated with worse clinical and patient experience outcomes compared with other diagnostic routes; these poorer outcomes are partially explained by later stage at diagnosis and disease-related complications.^1^^–^5 Welcome reductions in the proportion of patients with cancer who are diagnosed as emergencies have been reported,^6^ but there is uncertainty about the responsible mechanisms involving tumour, patient and healthcare system factors, and how to achieve further reductions.^7^^,^^8^

Diagnostic processes leading to emergency presentations can involve general practice in two different ways. First, about two-thirds of all patients with cancer who are diagnosed as emergencies would have had prior GP consultations with relevant symptoms, often leading to investigations or referrals.^9^^,^^10^ Second, GPs can be involved in the emergency presentation care episode itself. In England about one-third of all emergency presentations involve an emergency referral to hospital by a GP.^1^ In this article the authors focus on the latter aspect of general practice involvement in emergency presentations.

In England, the frequency of emergency presentations (denoting diagnosis of cancer following an emergency hospital admission or outpatient appointment) is routinely monitored through the ‘Routes to Diagnosis’ programme of the National Cancer Registration and Analysis Service (NCRAS) of Public Health England.^2^^,^^11^ Emergency presentations comprise different pathways (hereafter termed ‘sub-routes’), chiefly either emergency GP referral (GP-EP) or presentation to the accident and emergency (A&E) department (AE-EP).^12^^,^^13^ As these two sub-routes reflect different patterns of healthcare utilisation before a cancer diagnosis, understanding associated factors can elucidate different mechanisms and pathways, particularly regarding the role of general practice.^9^^,^^10^^,^^14^ Some of these pathways will represent appropriate care, for example, an emergency GP referral following presentation with symptoms or signs highly suggestive of cancer in an unwell patient, but others may reflect patient factors, for example, relatively late help seeking, or healthcare factors, for example, use of A&E departments due to difficulties in accessing primary care.^8^

**Table:** How this fits in

Primary care has a crucial, though often misrepresented, role in the diagnosis of cancer in symptomatic patients. There have been welcome declines in the proportion of cancers diagnosed as emergencies but reasons are not well understood. The authors observed declining numbers of patients presenting as emergencies of a specific type, that is, those generated after a GP has referred a patient to hospital as an emergency. Reductions in the number of emergency presentations likely reflect continually increasing 2-week-wait GP referrals during the study period as well as reductions in emergency presentations following a GP referral.

This study aims to characterise sociodemographic, cancer site, and temporal associations with emergency presentation sub-routes among patients with cancer diagnosed as emergencies. The objective was to examine patient groups that are either over- or under-represented in emergency presentations directly involving a GP emergency referral to hospital, and establish related temporal trends and their likely contribution to overall changes in how patients with cancer are being diagnosed.

## METHOD

### Diagnostic routes data

The authors studied Routes to Diagnosis data for 2006–2015 on patients aged ≥25 years diagnosed with any of 35 common and rarer cancers, responsible for 95% of incident cases. The ‘diagnostic route’ of each registered tumour is assigned by NCRAS using a rules-based (algorithmic) approach, which incorporates information from linked Hospital Episode Statistics, Cancer Waiting Times, and NHS Cancer Screening Programme data.^2^ The authors focused on patients diagnosed with cancer through an emergency presentation, defined in Routes to Diagnosis data as diagnosis of cancer during or after an emergency hospital admission (including via GP, A&E, or bed bureau) or A&E department attendance, including through direct presentation or after GP referral. The principal outcome of interest was emergency presentation sub-route, denoting different patterns of healthcare utilisation preceding the emergency cancer diagnosis. These included GP-EP (diagnosis during or after a hospital admission resulting from an emergency GP referral), AE-EP (diagnosis during or after a hospital admission following presentation to A&E), and Other-EP (diagnosis during a hospital admission not during or after an emergency GP referral, or presentation to A&E/dental casualty followed by hospital admission, for example, diagnosis during admission via bed bureau).

### Other variable data

Exposure variables included sex, age (grouped as aged 25–49, 50–59, 60–69, 70–79, and ≥80 years), social deprivation (five categories from least to most deprived, using quintile cut-offs for England of Index of Multiple Deprivation [income domain] scores based on residential postcode), cancer (35 different sites as defined by ICD-10 codes), and year of diagnosis.

### Statistical analyses

The analysis had two objectives. The first is to describe associations between exposure variables and each emergency presentation sub-route. The researchers reported the number and proportions (both crude and adjusted) of emergency presenters (*n* = 554 621) diagnosed via AE-EP, GP-EP, and Other-EP by sex, age group, deprivation status, cancer site, and year of diagnosis. Adjusted proportions were predicted from a multivariable multinomial logistic regression model where the outcome was AE-EP and Other-EP (baseline category: GP-EP) and all of the exposure variables were included as independent variables; reference categories: male, aged 70–79 years, least deprived, colon cancer, 2006.

The second objective was to describe temporal trends in each emergency presentation sub-route, also taking into account time trends across all other diagnostic routes.^6^ The authors presented the numbers of all incident cases of the studied cancers, that is, diagnosed through any route, including non-emergency presentation, over time, partitioned into adjusted numbers of each of the emergency presentation sub-routes and all other routes, using the same modelling technique as described above. These numbers were predicted from a second multinomial logistic regression model, this time including all cancer cases, as opposed to emergency presenters alone, *n* = 2 619 067, where the outcome was AE-EP, Other-EP, 2-week-wait (2WW) referral, non-emergency GP referral, screening, and ‘Other’ (baseline category: GP-EP), and including the same independent variables as in the first multinomial logistic regression model.

## RESULTS

Among the 554 621 patients presenting as emergencies during 2006–2015 (Table 1), 24% (*n* = 130 372) had presented through GP-EP, 62% (*n* = 346 192) had presented through AE-EP, and 14% (*n* = 78 057) through the Other-EP sub-route. Absolute numbers and crude and adjusted proportions of emergency presentations by GP-EP, AE-EP, and Other EP sub-routes, by sociodemographic variables, cancer site, and year of diagnosis are available from the authors on request.

**Table 1.** Number and adjusted proportions of emergency presentations by sociodemographic characteristic, cancer site, and year of diagnosis, stratified by GP-EP or AE-EP sub-routes (*N* = 554 621)

**Variable**	**Emergency cancer presentation**		

	**EP (all) *n***	**GP-EP Adjusted %^a^**	**AE-EP Adjusted %^a^**
**Sex**			
Female	262 173	24.0	61.8
Male	292 448	23.0	62.9

**Age, years**			
25–49	32 311	19.2	59.6
50–59	49 447	21.5	59.6
60–69	104 695	23.0	59.8
70–79	157 853	24.0	61.8
≥80	210 315	24.5	65.5

**Social deprivation quintiles**			
1 least deprived	91 724	25.1	59.6
2	108 860	26.2	59.6
3	114 391	25.2	61.2
4	118 498	22.7	64.1
5 most deprived	121 148	19.1	66.6

**Cancer type**			
Pancreatic	35 139	30.9	57.4
Ovarian	17 286	28.5	56.7
AML	12 283	28.9	54.6
ALL	1251	31.0	52.7
Colon	65 092	27.2	63.1
CUP	45 366	26.3	63.1
Gallbladder	3127	27.8	57.8
Sarcoma	3781	26.8	55.2
Oesophageal	14 505	26.3	62.6
CML	1741	26.4	58.7
Small intestinal	4746	25.9	63.1
Stomach	19 000	24.7	64.5
NHL	27 001	25.4	57.7
HL	2015	26.9	57.0
Multiple myeloma	14 349	24.2	58.3
Liver	9588	24.6	62.1
Anal	1230	24.4	62.7
Mesothelioma	7597	22.3	59.8
CLL	5694	21.3	64.8
Rectal	11 161	22.0	66.6
Bladder	15 989	20.2	63.0
Lung	128 938	20.9	66.6
Kidney	17 586	21.0	62.9
Prostate	31 485	19.3	61.1
Intracranial endocrine	149	21.6	51.1
Breast	18 199	18.7	66.3
Cervical	2565	19.6	65.6
Testicular	1456	23.2	53.2
Uterine	5528	17.4	61.6
Brain	22 361	17.2	60.2
Melanoma	2474	15.8	58.5
Thyroid	1493	14.5	57.5
Laryngeal	1904	12.3	68.7
Oropharyngeal	1221	13.4	59.2
Oral	1321	9.1	54.4

**Year**			
2006	56 104	31.0	55.3
2007	54 190	30.5	56.2
2008	55 421	27.9	59.1
2009	55 808	26.1	60.2
2010	54 450	23.8	62.1
2011	55 488	22.0	63.9
2012	56 713	20.2	65.1
2013	56 926	18.8	66.9
2014	55 379	18.0	67.1
2015	54 142	16.8	68.2

a *Estimated using a multinomial logistic regression model for AE-EP, GP-EP (reference outcome), and Other-EP, fitted to all patients diagnosed through EP in 2006 to 2015 (*N *= 554 621), where independent variables were sex, age group, deprivation group, cancer, and year of diagnosis. Proportions were predicted where each variable’s categories, for example, female and male, were forced to have the same case mix as that of the entire sample, 2006–2015. AE-EP = emergency presentation through presentation to accident and emergency. ALL = acute lymphoblastic leukaemia. AML = acute myeloid leukaemia. CLL = chronic lymphocytic leukaemia. CML = chronic myeloid leukaemia. CUP = cancer of unknown primary. GP-EP = emergency presentation through a GP referral. EP = emergency presentation HL = Hodgkin’s lymphoma. NHL = non-Hodgkin’s lymphoma. Other-EP = emergency presentation through routes other than through the GP or accident and emergency, for example, through referral during an inpatient admission.*

### Associations between sociodemographic characteristics and sub-route

There was limited variation in emergency presentation sub-route by sex. Patients who were older and presented as emergencies were more likely to be diagnosed both via GP-EP and via AE-EP compared with younger patients, reflecting that patients who were younger and presented as emergencies were more likely to be diagnosed via the Other-EP sub-route; adjusted proportion for those aged ≥80 years was 11% (*n* = 17 157) versus 21% (*n* = 7509) for ages 25–49 years (adjusted proportions for age groups for Other-EP are available from the authors on request). The likelihood of GP-EP decreased with increasing levels of deprivation, for example, adjusted proportions for least versus most deprived were 25% (*n* = 22 997) versus 19% (*n* = 22 775), while, in contrast, that of AE-EP increased (60%, *n* = 54493 versus 67%, *n* = 80 803) (corresponding percentage and number values for the remaining variables are available from the authors on request). There was little variation in the likelihood of Other-EP by levels of deprivation (15%, *n* = 14 234 versus 14%, *n* = 17 570). Given the large sample size, there was evidence (*P*<0.001) for variation in emergency presentation sub-route by variable category for all above variables (sex, age, deprivation group).

### Associations between cancer site and sub-route

Patients presenting as emergencies with pancreatic, acute myeloid and acute lymphoblastic leukaemia (ALL), ovarian, and gallbladder cancers were more likely to be diagnosed via the GP-EP sub-route (adjusted proportions of GP-EP ≥27%) (Table 1). In contrast, those diagnosed with oral, oropharyngeal, laryngeal, thyroid, melanoma, brain, and uterine cancers (adjusted proportions of GP-EP ≤18%) were least likely to be diagnosed via the GP-EP sub-route (Table 1). Notably, most cancer sites with low proportions of GP-EP, that is, oral, oropharyngeal, thyroid, melanoma, brain, and uterine cancers, had relatively high proportions of Other-EP. There was evidence (*P*<0.0001) for variation in emergency presentation sub-route by cancer site.

### Time trends

**Figure 1.**
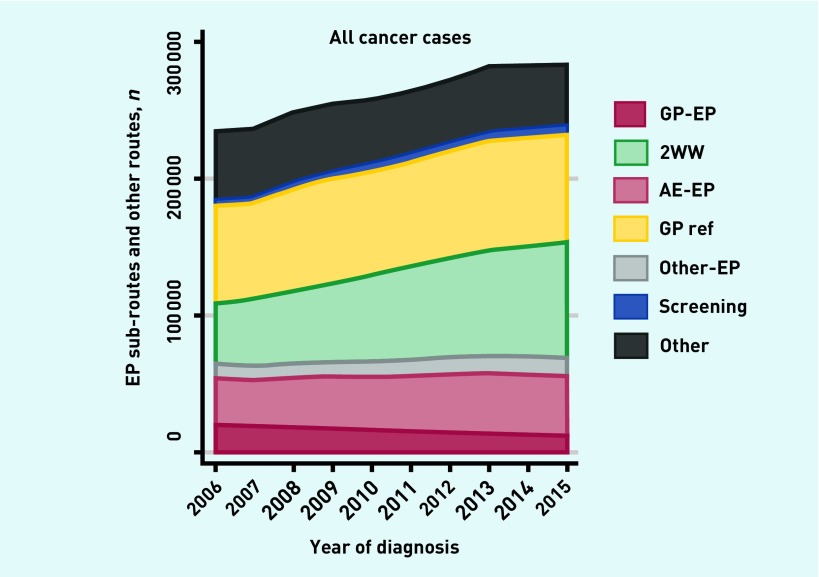
***Adjusted numbers of patients with cancer diagnosed via emergency presentation sub-route or any other diagnostic route, estimated using a multivariable multinomial logistic regression, for AE-EP, GP-EP (reference outcome), Other-EP, 2WW, non-emergency GP referral, screening, and ‘Other’, fitted to all patients diagnosed with cancer (including non-EP), 2006–2015 (*N *= 2 619 067), where independent variables were sex, age group, deprivation group, cancer, and year of diagnosis. Predicted numbers of AE-EPs, GP-EPs, Other-EPs, and non-EPs were derived by multiplying predicted proportions of these outcomes by the number of observed cancer cases (including non-EP), per year. AE-EP = emergency presentation through presentation to accident and emergency. EP = emergency presentation. GP-EP = emergency presentation through a GP referral. GP ref = non-emergency GP referral. Other-EP = emergency presentation through routes other than through the GP or accident and emergency, for example, through referral during an inpatient admission. 2WW = 2-week-wait referral.***

The number of incident cases for the studied cancers, adjusted for changes over time in sociodemographic characteristics and cancer site distributions, increased each year, for example, from 237 799 cases in 2006 to 284 660 cases in 2015, an increase of 20%, while the number of emergency presentations each year decreased slightly (from 56 104 to 54 142 respectively, a decrease of 4%; Table 1). Consequently, there was a progressive reduction in the adjusted number of cancers diagnosed as emergency presentations, and an expansion in the number of cancers diagnosed as non-emergency presentations, particularly via 2WW referrals (Figure 1). There was evidence (*P*<0.0001) for variation in emergency presentation sub-route by year of diagnosis.

**Figure 2.**
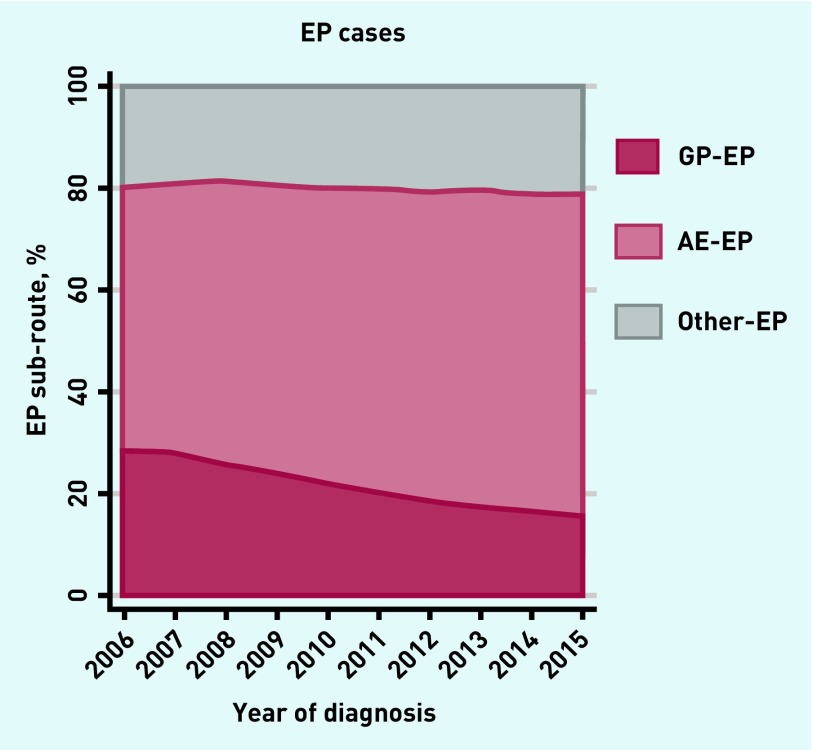
***Adjusted proportions of emergency presentation type among emergency presenters, over time, estimated using a multivariable multinomial logistic regression, for AE-EP, GP-EP (reference outcome), and Other-EP, fitted to all patients diagnosed through EP in 2006–2015 (*n *= 554 621), where independent variables were sex, age group, deprivation group, cancer, and year of diagnosis. AE-EP = emergency presentation through presentation to accident and emergency. EP = emergency presentation. GP-EP = emergency presentation through a GP referral. Other-EP = emergency presentation through routes other than through the GP or accident and emergency, for example, through referral during an inpatient admission.***

The changing proportion of emergency presentations overall was accompanied by a changing composition of EP sub-routes (Figure 2). GP-EP was less common among patients presenting as emergencies in more recent years of diagnosis (adjusted proportions down from 31%, *n* = 17 364, to 17%, *n* = 9155, between 2006 and 2015), while the opposite was true for AE-EP (up from 55%, *n* = 31 049 to 68%, *n* = 36 868; Table 1). The proportions of emergency presentation diagnosed through Other-EP slightly increased during this time (from 14%, *n* = 7691 to 15%, *n* = 8119). Relatedly, against a slight overall decrease in the absolute numbers of emergency presentations of any type between 2006 and 2015 (Table 1), the absolute numbers of GP-EPs almost halved (*n* = 17 364 to *n* = 9155) and that of AE-EPs slightly increased (*n* = 31 049 to *n* = 36 868). Absolute numbers and crude adjusted proportions of emergency presentations by GP-EP, AE-EP, and Other EP sub-routes for all years of diagnosis are available from the authors on request.

## DISCUSSION

### Summary

Over a recent decade, and against an overall continuous decrease in the percentage of patients diagnosed with cancer through an emergency presentation (from 24% in 2006 to 20% in 2015), the likelihood of emergency presentation via GP referral decreased, coupled with an increased likelihood of emergency presentation via A&E, even after accounting for sociodemographic and cancer site case mix over time. Among patients presenting as emergencies, patients living in areas of greater deprivation were less likely to be diagnosed via the GP-EP sub-route, and more likely to be diagnosed via the AE-EP sub-route. There was large heterogeneity in sub-route profile by cancer site.

### Strengths and limitations

The authors used a large population-based dataset covering a 10-year period and presented adjusted proportions of emergency presentation sub-routes by each patient characteristic, cancer site, and year of diagnosis, respectively. All variables included in this study come from high-quality cancer registration data. Diagnostic route, used to capture emergency presentation status and sub-route type, is derived via an algorithmic approach using linked routine population-based datasets.^2^

As common in observational studies, other variables (not available for inclusion in the analysis) could at least partially account for some of the reported sociodemographic, cancer site, and temporal variation in emergency presentation sub-routes. It could be revealing to examine the potential interplay between stage at diagnosis and emergency presentation, as advanced stage is associated with greater likelihood of emergency presentation,^3^ but the authors could not address this question within the current study owing to poor completeness of stage at diagnosis information during most of the study years, 2006–2015.^15^^–^17

A limitation inherent to all research using routine data is that it does not allow for exact circumstances to be taken into account. For example, a patient presenting for emergency may have attended A&E following verbal GP advice without a formal referral. This hypothetical patient would have likely been assigned an AE-EP sub-route, while GP-EP sub-route may have been more apt, which would introduce misclassification error. The magnitude of such a misclassification is difficult to quantify but is likely to be small.^9^^,^^10^

### Comparison with existing literature

There are no other population-based studies of emergency presentation sub-routes for cancer cases in England covering both common and rarer cancers with which to compare the present results.^8^ Nonetheless, this study builds on previous reports of crude proportions of emergency presentation sub-route by age and cancer site,^12^^,^^13^ by reporting proportions adjusted for patient case mix (age, sex, deprivation, and cancer site). A previous study focusing on patients with lung cancer found that GP-EP sub-route was least likely in patients presenting as emergencies who were more deprived, and vice versa for AE-EP,^18^ concordant with the present study, which covers a much wider range of cancer sites.

### Implications for research and practice

The findings overall indicate that certain cancers that can be associated with painless symptoms that may be tolerable to the patient but where GPs may appropriately request an urgent specialist assessment — for example, pancreatic or gallbladder cancer presenting with painless jaundice; acute leukaemia presenting with pallor; ovarian cancer presenting with abdominal distension — were associated with the highest likelihoods of GP-EP in this study. These findings underline the importance of tumour factors as a contributor to emergency presentations in some patients. As these symptoms have relatively high predictive values for cancer,^19^ they can lead to appropriately accelerated assessment through ‘same- or next-day’ clinics, thus explaining the increased likelihood of diagnosis via GP-EPs that nevertheless represent good GP care. Some cancers had particularly high proportions of Other-EP, including oral, oropharyngeal, thyroid, melanoma, and brain cancer. This likely denotes either the involvement of other clinical specialties, for example, dental practitioners in the case of oral/oropharyngeal cancers, or greater than average involvement of hospital department clinics in the diagnosis of certain cancers. Patients who were younger and presented as emergencies were also more likely to be diagnosed through Other-EPs than patients who were older and presented as emergencies. As suspecting the diagnosis of cancer in patients who are younger is typically harder than in those who are older, they may be more likely to be initially referred to specialist clinics, and progress to an emergency presentation through these clinics.^20^^,^^21^

As more deprived patients presenting as emergencies were less likely to have been referred by their GP and more likely to have presented to A&E, help-seeking patterns among otherwise similar patients subsequently diagnosed with the same cancer seem to vary by socioeconomic status.

Against an overall decreasing proportion of patients with cancer who are diagnosed through emergency presentations, the number of patients diagnosed through the GP-EP sub-route is decreasing, while that of patients diagnosed through AE-EP and non-emergency routes is increasing. This decreasing trend in emergency presentations overall has occurred in spite of other evidence for opposite, (increasing) trends in both general A&E attendance and emergency hospital admissions.^22^^–^27 Therefore, the overall trend cannot be accounted for by such general A&E/emergency admission trends, and likely reflects a reciprocal rise in the use by GPs of 2WW referrals for suspected cancer in the same period (Figure 1).^28^ Among emergency presenters, reductions in the GP-EP sub-route could reflect increasing difficulties in accessing in-hours primary care among patients with possible cancer symptoms;^26^^,^^29^^,^^30^ and the progressive shrinkage (through the overall increase in 2WW referrals) of the pool of patients who would have otherwise been diagnosed with cancer as an emergency presentation.

## References

[1] Pham TM, Gomez-Cano M, Salika T (2019). Diagnostic route is associated with care satisfaction independently of tumour stage: Evidence from linked English Cancer Patient Experience Survey and cancer registration data. Cancer Epidemiol.

[2] Elliss-Brookes L, McPhail S, Ives A (2012). Routes to Diagnosis for cancer — determining the patient journey using multiple routine data sets. Br J Cancer.

[3] McPhail S, Elliss-Brookes L, Shelton J (2013). Emergency presentation of cancer and short–term mortality. Br J Cancer.

[4] National Cancer Registration and Analysis Service. (2018). Routes and treatment. Tumour resections, chemotherapy and radiotherapy by Route, cancer site and patient characteristics, England, 2013–2015.

[5] Salika T, Abel GA, Mendonca SC (2018). Associations between diagnostic pathways and care experience in colorectal cancer: evidence from patient-reported data. Frontline Gastroenterol.

[6] Herbert A, Abel GA, Winters S (2019). Are inequalities in cancer diagnosis through emergency presentation narrowing, widening or remaining unchanged? Longitudinal analysis of English population–based data 2006–2013. J Epidemiol Community Health.

[7] Lyratzopoulos G, Saunders CL, Abel GA (2014). Are emergency diagnoses of cancer avoidable? A proposed taxonomy to motivate study design and support service improvement. Future Oncol.

[8] Zhou Y, Abel GA, Hamilton W (2017). Diagnosis of cancer as an emergency: a critical review of current evidence. Nat Rev Clin Oncol.

[9] Abel GA, Mendonca SC, McPhail S (2017). Emergency diagnosis of cancer and previous general practice consultations: insights from linked patient survey data.. Br J Gen Pract.

[10] Murchie P, Smith SM, Yule MS (2017). Does emergency presentation of cancer represent poor performance in primary care? Insights from a novel analysis of linked primary and secondary care data. Br J Cancer.

[11] National Cancer Registration and Analysis Service. (2019). Routes to Diagnosis. http://www.ncin.org.uk/publications/routes_to_diagnosis.

[12] National Cancer Registration and Analysis Service. (2019). Routes to Diagnosis 2006–2016 workbook (b).

[13] National Cancer Intelligence Network. (2013). Routes to Diagnosis: exploring emergency presentations.

[14] Renzi C, Lyratzopoulos G, Card T (2016). Do colorectal cancer patients diagnosed as an emergency differ from non-emergency patients in their consultation patterns and symptoms? A longitudinal data-linkage study in England. Br J Cancer.

[15] Benitez-Majano S, Fowler H, Maringe C (2016). Deriving stage at diagnosis from multiple population-based sources: colorectal and lung cancer in England. Br J Cancer.

[16] United Kingdom and Ireland Association of Cancer Registries. (2017). United Kingdom and Ireland Association of Cancer Registries (UKIACR) performance indicators 2017 report..

[17] National Cancer Registration and Analysis Service. (2015). Routes to Diagnosis of cancer by stage, 2012–2013..

[18] Maringe C, Pashayan N, Rubio FJ (2018). Trends in lung cancer emergency presentation in England, 2006–2013: is there a pattern by general practice?. BMC Cancer.

[19] Stapley S, Peters TJ, Neal RD (2012). The risk of pancreatic cancer in symptomatic patients in primary care: a large case-control study using electronic records. Br J Cancer.

[20] Dommett RM, Redaniel MT, Stevens MCG (2013). Features of cancer in teenagers and young adults in primary care: a population-based nested case–control study. Br J Cancer.

[21] Fern LA, Birch R, Whelan J (2013). Why can’t we improve the timeliness of cancer diagnosis in children, teenagers, and young adults?. BMJ.

[22] Blunt I, Bardsley M, Dixon J (2010). Trends in emergency admissions in England 2004–2009: is greater efficiency breeding inefficiency?.

[23] Cowling TE, Soljak MA, Bell D, Majeed A (2014). Emergency hospital admissions via accident and emergency departments in England: time trend, conceptual framework and policy implications. J R Soc Med.

[24] Hull SA, Homer K, Boomla K (2018). Population and patient factors affecting emergency department attendance in London: retrospective cohort analysis of linked primary and secondary care records.. Br J Gen Pract.

[25] Cancer Research UK (2017). Be clear on cancer.. http://www.cancerresearchuk.org/health-professional/early-diagnosis-activities/be-clear-on-cancer.

[26] Cowling TE, Harris MJ, Watt HC (2014). Access to general practice and visits to accident and emergency departments in England: cross-sectional analysis of a national patient survey.. Br J Gen Pract.

[27] Scantlebury R, Rowlands G, Durbaba S (2015). Socioeconomic deprivation and accident and emergency attendances: cross-sectional analysis of general practices in England.. Br J Gen Pract.

[28] Public Health England. (2016). Trends in cancer waiting times metrics, England, 2009/10 to 2014/15..

[29] British Medical Association. (2018). Analysis of GP Patient Survey 2017..

[30] Hobbs FDR, Bankhead C, Mukhtar T (2016). Clinical workload in UK primary care: a retrospective analysis of 100 million consultations in England, 2007–14. Lancet.

